# Mutant Prevention Concentration, Frequency of Spontaneous Mutant Selection, and Mutant Selection Window—a New Approach to the *In Vitro* Determination of the Antimicrobial Potency of Compounds

**DOI:** 10.1128/aac.01373-22

**Published:** 2023-04-06

**Authors:** Joanna Krajewska, Stefan Tyski, Agnieszka E. Laudy

**Affiliations:** a Department of Pharmaceutical Microbiology and Bioanalysis, Medical University of Warsaw, Warsaw, Poland; b Department of Antibiotics and Microbiology, National Medicines Institute, Warsaw, Poland

**Keywords:** MPC, MSW, FSMS, MPC-D, MSW-D, MPC-F, MSW-F, antibiotic resistance, antimicrobial activity, antimicrobial agents

## Abstract

The analysis of antimicrobial activity is usually MIC- and minimal bactericidal concentration (MBC)-focused, though also crucial are resistance-related parameters, e.g., the frequency of spontaneous mutant selection (FSMS), the mutant prevention concentration (MPC), and the mutant selection window (MSW). *In vitro*-determined MPCs, however, are sometimes variable, poorly repeatable, and not always reproducible *in vivo*. We propose a new approach to the *in vitro* determination of MSWs, along with novel parameters: MPC-D, MSW-D (for dominant mutants, i.e., selected with a high frequency, without a fitness loss), and MPC-F, MSW-F (for inferior mutants, i.e., with an impaired fitness). We also propose a new method for preparing the high-density inoculum (>10^11^ CFU/mL). In this study, the MPC and MPC-D (limited by FSMS of <10^−10^) of ciprofloxacin, linezolid, and novel benzosiloxaborole (No37) were determined for Staphylococcus aureus ATCC 29213 using the standard agar method, while the MPC-D and MPC-F were determined by the novel broth method. Regardless of the method, MSWs_10_^10^ of linezolid and No37 were the same. However, MSWs_10_^10^ of ciprofloxacin in the broth method was narrower than in the agar method. In the broth method, the 24-h incubation of ~10^10^ CFU in a drug-containing broth differentiates the mutants that can dominate the cell population from those that can only be selected under exposure. We consider MPC-Ds in the agar method to be less variable and more repeatable than MPCs. Meanwhile, the broth method may decrease discrepancies between *in vitro* and *in vivo* MSWs. The proposed approaches may help establish MPC-D-related resistance-restricting therapies.

## INTRODUCTION

*In vitro* parameters describing the antimicrobial potency of compounds are important concerning the increasing antimicrobial resistance. They apply to the search for both new combination therapies (known antibiotics that would show a synergistic effect) and structurally new compounds. Based on these parameters, the best new drug candidate is selected, while many other compounds are eliminated from further studies. Currently, despite tremendous efforts and an abundance of compounds in preclinical trials, the number of new antibiotics on the market is still insufficient (1 to 4). Thus, refining old or introducing new *in vitro* parameters (especially those that can predict the emergence and the expansion of resistant mutants) is an approach aimed at improving the preclinical screening for novel antimicrobials and, consequently, facilitating the development of novel therapies.

Currently, the most common parameters determined in preliminary studies concerning the activity of newly developed compounds against microorganisms are the MIC values and the minimal bactericidal/fungicidal concentration (MBC/MFC) values (5 to 8). However, an extremely important stage of preclinical research is also to determine the resistance-related parameters like the frequency of spontaneous mutant selection (FSMS) and the so-called mutant prevention concentration (MPC) values (9 to 12). These are necessary for subsequent *in vivo* studies to determine the dose of the new compound as a drug in animal models. It is known that resistant cells can be selected at a high frequency in the population of bacteria living in the presence of an antibiotic in a concentration ranging from the MIC value to slightly above the MIC value (9, 10). In the natural environment, e.g., in a human host, a single-step mutation often occurs in bacterial cells. If the frequency of spontaneous mutations is very low, the host’s immune system will likely be able to combat the emerging bacterial mutants (10). Zhao et al., in their reviews on the restriction of fluoroquinolone-resistant mutant selection in Staphylococcus aureus and Mycobacterium bovis cell population, indicated that mutants arising at a low FSMS value of 1 × 10^−6^ to 1 × 10^−8^ could be controlled by the host organism (10, 11). Similarly, Sun et al. used a frequency of <1 × 10^−8^ as a threshold for reduced mutant selection in *in vitro* analysis assessing the activity of the meropenem–vaborbactam combination against Klebsiella pneumoniae strains producing the Klebsiella pneumoniae carbapenemase (KPC)-type enzymes (13). Moreover, mutants with a significant fitness cost of acquired resistance (with the decreased growth rate) also cannot establish a resistant population *in vivo* and can be easily outcompeted by sensitive bacteria in a nonantibiotic environment (14, 15). On the other hand, in preclinical *in vitro* studies of new compounds, the concept of MPC was introduced as a value relating to the prevention of the emergence of resistant mutants’ growth. The concept and definition of MPC were proposed by Dong et al. in 1999 (9). According to this first definition, the MPC is estimated by determining the minimal antibiotic concentration that allows no mutant to be obtained when a large number of bacterial cells (>10^10^) are applied to agar plates containing the antibiotic. Thus, the MPC value is sufficient to block the growth of single-step mutants (9 to 11). More importantly, Dong et al. underlined that the MPC value depends on the number of cells used in the study (9). Consequently, a subscript indicating the number of cells used in the test should be added to the MPC (e.g., MPC_10_^10^ indicates that 10^10^ cells were applied to the plates). The acquired antibiotic resistance of the obtained mutants was checked by their regrowth on the new agar plates with the same concentration of antibiotic (9).

The selective proliferation of resistant mutants (also called mutants enrichment) is considered to occur only in the presence of an antibiotic in the concentration range from the MIC value for a wide-type strain up to the MPC value that inhibits the growth of single-step resistant mutants. This interval of the antibiotic concentrations was named the mutant selection window (MSW) (10). However, it is also known that mutants may arise in sub-MIC concentrations of antibiotics (16 to 19), though their recovery drops off sharply then (20), as the growth of sensitive wide-type cells is not inhibited (10). The basis for determining the currently used drug doses are the MIC values and the standardized cutoff points defining the susceptibility or resistance of the tested strains, according to the European Committee on Antimicrobial Susceptibility Testing (EUCAST) (21) and the Clinical and Laboratory Standards Institute (CLSI) documents (22). The MPC/MIC ratio ranges from 4 to >32, depending on the antibiotic and the tested strain (9, 23 to 27). It seems that such a sizeable x-fold increase in drug dose is not always necessary (20, 28) and not always feasible, due to a higher risk of side effects, with little benefit to the patient (10), especially since MIC-based doses are generally high enough to clear the infection with the lowest possible toxicity (10). On the other hand, the problem may occur with immunocompromised patients who may experience therapeutic failure due to the generation of resistant mutants, as emphasized by Zhao and Drlica (10). Another problem is the scale of the mutant selection in a public health context. When the bulk of therapies put antibiotic concentrations within the MSW, the abundance of selected mutants and their spread will accelerate the loss of its activity (10). Therefore, the question may still be raised about what concentrations should be used to achieve therapeutic success, minimize toxicity for the patients and, at the same time, not generate a selection of bacterial mutants to preserve the effectiveness of antibiotics.

In this article, we propose a new approach to *in vitro* determination of the resistance potential of antibacterial compounds. To date, no MPC-based, resistance-restricting dosing scheme is in use. Moreover, Gianvecchio et al. have recently reported poor repeatability and reproducibility of MPC results for some strain-drug combinations (29), while others observed some discrepancies between the ranges of *in vitro*- and *in vivo*-determined MSWs (30, 31). We assume that this is related to the frequency of mutant selection. Mutants selected extremely rarely may not always be detectable in the agar-dilution method. Another problem is the mutants’ fitness, which is not assessed during the MPC determination. Considering the above, we propose the following new resistance-related parameters characterizing the *in vitro*-determined antimicrobial potency of compounds: a dominant mutant prevention concentration (MPC-D), an inferior mutant prevention concentration (MPC-F), a dominant mutant selection window (MSW-D), and an inferior mutant selection window (MSW-F). MPC-D and MSW-D refer to mutants selected with a high frequency, without a resistance-associated fitness loss, which we named the dominant mutants. We assume they are likely to establish a resistant population *in vivo* and should be considered relevant in the public health context. MPC-D is the lowest drug concentration that blocks the selection of dominant mutants. In the agar-dilution method, we defined MPC-D as the lowest drug concentration with the FSMS <10^−10^, considering mutants selected less often are hardly detectable and of less significance due to their rarity. However, to quickly predict whether mutants selected *in vitro* are not significantly inferior to sensitive cells due to the fitness costs of the resistance, we proposed a new broth-dilution method for their selection. In this method, we defined MPC-D as the lowest drug concentration that, after the 24 h of incubation, prevents mutants selected among 10^10^ CFU from establishing a resistant population of at least 10 CFU/mL in the drug-supplemented broth culture (i.e., mutants are not able to dominate the population). In turn, MPC-F and MSW-F refer to mutants with impaired fitness that can be selected *in vitro* in concentrations above the MPC-D but cannot dominate the population in the broth culture. We named them the inferior mutants, whereas MPC-F was defined as the lowest drug concentration that blocks their selection. We consider mutants unable to dominate in the broth culture unlikely to appear in subsequent *in vivo* studies. The assumptions of our work were based on the original definition of MPC. However, we believe that the ability of mutants to dominate the cell population, rather than mutant selection, is the most important point to consider in the further steps of both *in vivo* animal studies and clinical trials.

## RESULTS

### Susceptibility testing and the high-density inoculum.

The MIC values for the parent strain of Staphylococcus aureus ATCC 29213 were as follows: 0.25 mg/L for ciprofloxacin, 2 mg/L for linezolid, and 3.12 mg/L for novel benzosiloxaborole No37.

The assumed high-density inoculum (>10^11^ CFU/mL) was obtained by a 6-stage progressive culture concentration by centrifugation. Obtained densities for the two repetitions of the experiment were 7.5 × 10^11^ CFU/mL and 5 × 10^11^ CFU/mL.

### Frequency of spontaneous mutant selection (FSMS).

The single-step spontaneous mutant selection was performed on A-series plates (mutant selection by the agar-dilution method [AM]), which were visually assessed after incubation. Four types of growth were observed on the plates, depending on the tested antimicrobial compound and its concentration (Table 1).

**TABLE 1:** Agent-resistant mutant selection and their ability to regrow on the same concentration of the antibacterial compound

Agent tested^*a*^	Agent concentration		Agar-dilution method: MPC and MPC-D determination^*b*^		FSMS^*b*^	Broth-dilution method: MPC-D determination^*b*^			Broth-dilution method: MPC-F determination^*b*^		
	mg/L	x-fold MIC	Visual mutants’ growth on A-series plates^*c*^	aMRTests’ results (hours to mutants’ regrowth)^*b*^		Visual mutants’ growth on B-series plates^*c*^	bMRTests’ results (hours to mutants’ regrowth)^*b*^		Visual mutants’ growth on C-series plates^*c*^	bMRTests’ results (hours to mutants’ regrowth)^*b*^	
				Agar medium			Broth medium	Agar medium		Broth medium	Agar medium
CIP	0.25	1	L	+(24)	>3 × 10^−8^	L	+(24)	+(24)	L	+(24)	+(24)
	0.5	2	L	+(24)	>3 × 10^−8^	CL	+(24)	+(24)	CL	+(24)	+(24)
	1	4	CL	+(24)	>3 × 10^−8^	65	+(24)	+(24)	CL	+(24)	+(24)
	2	8	330	+(24)	5,5 × 10^−9^	NL	−(72)	−(72)	25	+(24)	+(24)
	4	16	4	+(24)	6 × 10^−11^	NL	−(72)	−(72)	1	+(24)	+(24)
	8	32	3	+(24)	4,5 × 10^−11^	NL	−(72)	−(72)	NL	−(72)	−(72)
LIN	2	1	L	+(24)	>3 × 10^−8^	L	+(24)	+(24)	L	+(24)	+(24)
	4	2	L	−(72)	<2 × 10^−11^	SL	−(72)	−(72)	L	−(72)	−(72)
	8	4	L	−(72)	<2 × 10^−11^	NL	−(72)	−(72)	NL	−(72)	−(72)
	16	8	SL	−(72)	<2 × 10^−11^	NL	−(72)	−(72)	NL	−(72)	−(72)
	32	16	SL	−(72)	<2 × 10^−11^	NL	−(72)	−(72)	NL	−(72)	−(72)
	64	32	SL	−(72)	<2 × 10^−11^	NL	−(72)	−(72)	NL	−(72)	−(72)
No37	3.12	1	L	+(24)	>3 × 10^−8^	L	+(24)	+(24)	L	+(24)	+(24)
	6.24	2	L	+(24)	>3 × 10^−8^	L	+(24)	+(24)	L	+(24)	+(24)
	12.5	4	L	+(24)	>3 × 10^−8^	CL	+(24)	+(24)	L	+(24)	+(24)
	25	8	L	+(48)	>3 × 10^−8^	CL	+(48)	+(48)	L	+(48)	+(48)
	50	16	L	+(48)	>3 × 10^−8^	CL	+(48)	+(72)	L	+(48)	+(72)
	100	32	L	−(72)	<2 × 10^−11^	NL	−(72)	−(72)	CL	−(72)	−(72)

a CIP, ciprofloxacin; LIN, linezolid; No37, novel benzosiloxaborole compound No37.

b MPC, mutant prevention concentration; MPC-D, dominant mutant prevention concentration; MPC-F, inferior mutant prevention concentration; FSMS, frequency of spontaneous mutant selection; aMRTests, agar mutants recovery tests; bMRTests, broth mutants recovery tests.

c L, dense lawn; SL, semi-lawn (less dense, no colonies visible); CL, colony lawn (uncountable colonies); NL, no lawn (visually clear plate).

In the case of ciprofloxacin, the dense bacterial lawn was detected at concentrations up to 0.5 mg/L (2×MIC), the colony lawn with uncountable colonies at the concentration of 1 mg/L (4×MIC), and countable colonies were detected at every higher concentration. All obtained colonies were recovered on agar medium in the agar mutant recovery test (aMRTest). Therefore, colony numbers at concentrations up to 1 mg/L (4×MIC) were classified as uncountable, and the FSMS values were classified as being above the upper detection limit (>3 × 10^−8^). Colonies at the 2-, 4-, and 8-mg/L concentrations were counted (mean values are presented in Table 1), and the FSMS values were calculated as 5.5 × 10^−9^, 6 × 10^−11^, and 4.5 × 10^−11^, respectively (means from two repetitions).

Visual screening of the linezolid A-series plates only revealed a dense, noncolony lawn, slightly thinner on the three highest concentrations tested. Such a result made colony counting impossible. In the aMRTest, however, mutant growth was only confirmed for the concentration equal to 2 mg/L (1×MIC), although no visual difference in the density of the plate lawns was observed for concentrations up to 4×MIC. Based on the aMRTest results, the FSMS for 2 mg/L (1×MIC) was established as being above the upper detection limit (>3 × 10^−8^). In contrast, those for concentrations of 4 mg/L and above (≥2×MIC) were considered as being below the lower limit of detection (<2 × 10^−11^).

The aMRTest was also required to determine the FSMS values for different concentrations of compound No37, as only a dense, noncolony lawn was observed on all A-series plates, regardless of the concentration. The aMRTest revealed that mutants were present on all plates except those with the highest concentration tested (100 mg/L, 32×MIC); however, the regrowth of mutants selected at No37 concentrations of 25 mg/L (8×MIC) and 50 mg/L (16×MIC) was impaired. At 8×MIC and 16×MIC, mutants needed 48 h to recover on agar. Based on the aMRTest results, the FSMS for concentrations equal to and below 50 mg/L (≤16×MIC) were calculated as being above the upper detection limit (>3 × 10^−8^). In comparison, those for the concentration of 100 mg/L (32×MIC) were considered being below the lower limit of detection (<2 × 10^−11^).

Reduced mutant selection (<10^−8^) was obtained for concentrations ≥8×MIC for ciprofloxacin, ≥2×MIC for linezolid, and ≥32×MIC for compound No37.

### Mutant prevention concentration (MPC) value and dominant mutant prevention concentration (MPC-D) parameter, determined by the agar-dilution method (AM).

The visual mutants’ growth on A-series plates, the mutants’ ability to regrow in the aMRTest, and the cutoff points for both the MPC and MPC-D parameters are presented in Table 1, while the MPC_10_^10^, MPC-D_10_^10^, selection index (SI), and dominant selection index (SI-D) values for the tested agents are shown in Table 2.

**TABLE 2:** Activity of tested agents, expressed as MPC and SI values

Methods	Parameters of activity^*a*^	Agents^*b*^		
		CIP	LIN	No37
Susceptibility test	MIC [mg/L]	0.25	2	3.12
Agar dilution	MPC_10_^10^ [mg/L]	>8	4	100
	SI	>32	2	32
	MPC-D_10_^10^ [mg/L]	4	4	100
	SI-D	16	2	32
Broth dilution	MPC-D_10_^10^ [mg/L]	2	4	100
	SI-D	8	2	32
	MPC-F_10_^10^ [mg/L]	8	4	100
	SI-F	32	2	32

a MPC, mutant prevention concentration; SI, selection index; MPC-D, dominant mutant prevention concentration; SI-D, dominant selection index; MPC-F, inferior mutant prevention concentration; SI-F, inferior selection index.

b CIP, ciprofloxacin; LIN, linezolid; No37, novel benzosiloxaborole compound No37.

In the case of ciprofloxacin, as mutants were also detected at the highest tested concentration of 8 mg/L (32×MIC), the MPC_10_^10^ value was estimated as being above 8 mg/L (>32×MIC). However, we consider mutants selected with extremely rare frequency (<1 × 10^−10^) unlikely to appear later *in vivo* often enough to be clinically significant (thus, they are not dominant mutants). Consequently, 4 mg/L (16×MIC) was designated the MPC-D_10_^10^ of ciprofloxacin, as it was the lowest concentration with the FSMS value below 1 × 10^−10^.

On the contrary, when linezolid and compound No37 were tested, a visual assessment of mutant presence on the plates was impossible: only a gradually fading lawn was observed on all A-series plates with linezolid, while a dense lawn was present on all plates for compound No37, including the highest tested concentration. The aMRTest revealed mutants capable of regrowth in the presence of the tested agent and allowed the MPC_10_^10^ values to be determined as 4 mg/L (2×MIC) for linezolid and 100 mg/L (32×MIC) for compound No37. Because a sharp decrease in the FSMS values (from >3 × 10^−8^ to <2 × 10^−11^) was shown between 1×MIC (2 mg/L) and 2×MIC (4 mg/L) for linezolid and between 50 mg/L (16×MIC) and 100 mg/L (32×MIC) for compound No37, the concentrations of 4 mg/L of linezolid and 100 mg/L of compound No37 were designated the MPC-D_10_^10^.

The MPC_10_^10^ values determined for two of the three compounds tested, linezolid and compound No37, were equal to the MPC-D_10_^10^ values, while the MPC_10_^10^ of ciprofloxacin was at least 2 times higher than the MPC-D_10_^10^ value associated with its FSMS.

Overall, linezolid had the smallest selection indices in the AM: its SI (MPC/MIC ratio) and SI-D (MPC-D/MIC ratio) were equal to 2.

### Dominant mutant prevention concentration (MPC-D) and inferior mutant prevention concentration (MPC-F) parameters, determined by the broth-dilution method (BM).

The visual mutants’ growth on B-series plates (inoculated with the aliquots from the liquid culture after 24 h of incubation) and C-series plates (inoculated with the concentrated content of the liquid culture after 24 h of incubation), results of the broth mutant recovery test (bMRTest), and the cutoff points for both MPC-D and MPC-F parameters are presented in Table 1, while the MPC-D_10_^10^, MPC-F_10_^10^, SI-D, and inferior selection index (SI-F) values for tested agents are shown in Table 2.

In the case of ciprofloxacin on B-series plates, a dense lawn was observed at 0.25 mg/L (1×MIC), an uncountable colony lawn at 0.5 mg/L (2×MIC), and 65 colonies were detected at 1 mg/L (4×MIC). No visual growth was observed on plates with 2, 4, and 8 mg/L of ciprofloxacin (8 to 32×MIC). The regrowth ability of the obtained resistant mutants in the presence of an antibiotic was confirmed by the bMRTest. Thus, the concentration of 2 mg/L (8×MIC) was designated the MPC-D_10_^10^ value. On ciprofloxacin C-series plates, a dense lawn was observed at 0.25 mg/L (1×MIC), an uncountable colony lawn at 0.5 to 1 mg/L (2 to 4×MIC), 25 colonies at 2 mg/L (8×MIC), and 1 colony at 4 mg/L (16×MIC). No colony was obtained on the plate with 8 mg/L (32×MIC) of ciprofloxacin, which is why this concentration was designated the MPC-F_10_^10^ for ciprofloxacin.

On B- and C-series linezolid plates, a dense lawn was observed at 2 mg/L (1×MIC), while at 4 mg/L (2×MIC) a thicker lawn, a semi-lawn, and a dense lawn, respectively. In the bMRTest, however, the bacterial lawn obtained at 4 mg/L was not able to recover in broth medium with the same linezolid concentration, which is why the MPC-D_10_^10^ and MPC-F_10_^10^ values were determined as 4 mg/L.

On B-series plates with compound No37, a dense lawn was visible up to 6.25 mg/L (2×MIC), while a colony lawn from 12.5 to 50 mg/L (4 to 16×MIC). No visual growth was observed at the concentration of 100 mg/L (32×MIC). The bMRTest confirmed obtained resistant mutants’ ability to regrow in the same antibiotic concentrations. Thus, the concentration of 100 mg/L (32×MIC) was designated the MPC-D_10_^10^ value. In contrast, on C-series plates, the colony lawn visible at 100 mg/L failed the bMRTest. As a result, the MPC-F_10_^10^ value was also designated 100 mg/L.

The MPC-D_10_^10^ values determined for two of the three compounds tested, linezolid and compound No37, were equal to the MPC-F_10_^10^ values, while the MPC-F_10_^10^ of ciprofloxacin was 4 times higher than the MPC-D_10_^10^ value.

Overall, in the broth-dilution method, linezolid had the smallest SI-D and SI-F (both equal to 2), followed by ciprofloxacin (SI-D = 8, SI-F = 32) and compound No37 (both equal to 32).

### Mutant selection window (MSW), dominant mutant selection window (MSW-D), and inferior mutant selection window (MSW-F) ranges.

**FIG 1:**
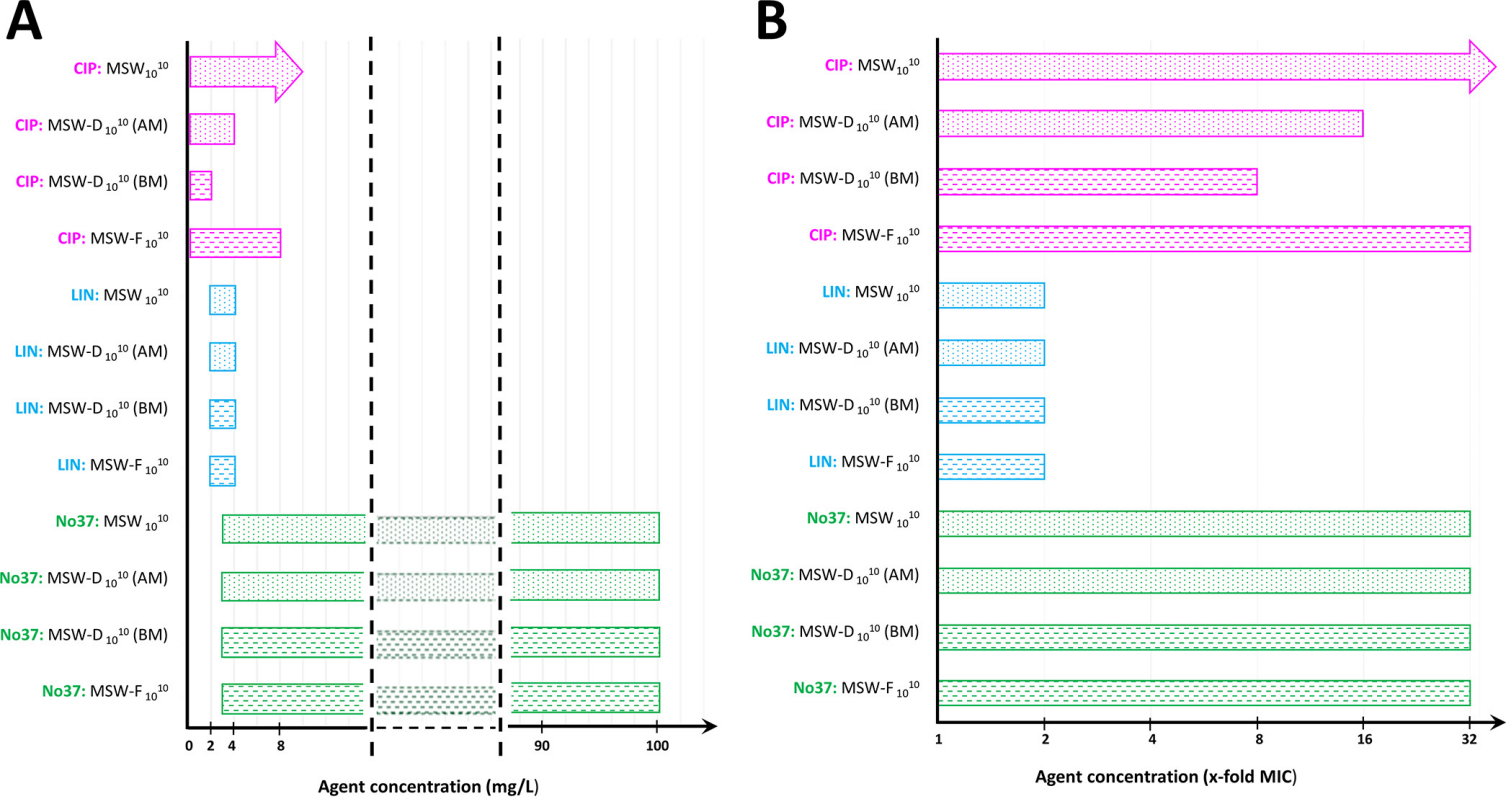
Scheme presenting the MSW_10_^10^ ranges. Agents’ concentrations expressed in mg/L (A) or as x-fold MIC (B). CIP, ciprofloxacin; LIN, linezolid; No37, novel benzosiloxaborole compound No37; MSW, mutant selection window; MSW-D, dominant mutant selection window; MSW-F, inferior mutant selection window; AM, agar-dilution method; BM, broth-dilution method; pink color, MSWs of CIP; blue color, MSWs of LIN; green color, MSWs of No37; dotted pattern, MSWs determined by the agar-dilution method; striped pattern, MSWs determined by the broth-dilution method; arrows indicate the MSW’s upper boundary was above the highest concentration tested.

The ranges of MSW, MPC-D, and MSW-F are presented in Fig. 1.

Overall, linezolid had the narrowest ranges of MSWs, MSW-Ds, and MSW-Fs. Regardless of the method, all of the above-mentioned parameters ranged between 1 and 2×MIC. Also, for compound No37, the MSW, MSW-Ds, and MSW-F had the same range of 1 to 32×MIC. In the case of ciprofloxacin, the narrowest range had the MSW-D_10_^10^ determined in the BM (1 to 8×MIC), while the MSW-D_10_^10^ determined in the AM was broader, ranging up to 4 mg/L (16×MIC), and the MSW-F_10_^10^ ranged up to 8 mg/L (32×MIC). The upper limit of the MSW_10_^10^ for ciprofloxacin was above the highest tested concentration of 8 mg/L (>32×MIC).

## DISCUSSION

Since the introduction of the mutant selection window hypothesis in 2001 (10), MPC values have been determined for a large number of isolates according to the initially proposed agar-dilution method (9). Generally, there is no correlation between MPC and MIC (32). Moreover, currently used doses were proven to put many drug concentrations inside the mutant selection window during therapy (11). However, for new fluoroquinolones, achieving serum concentrations above the MPC was possible with existing dosing schemes (33, 34). For compounds whose serum level cannot exceed the MPC (e.g., due to toxicity concerns), combination therapy was proved to restrict the mutant selection, as then, two concurrent mutations are needed for resistance (10, 11, 35). Finally, it was established that derivatives within the same group of antibacterial compounds might differ significantly in the mutant selection window size for particular strains (9, 11, 33, 34, 36 to 38). Consequently, preferential use of the derivatives with narrow selection windows may prolong the life span of the whole class of antibiotics (10). Though generally successful, the broad validation of the mutant selection window hypothesis provides some evidence that *in vitro*-determined MSW ranges do not always fit *in vivo* boundaries (30, 31). Also, concerns about the repeatability of the MPC values have been recently raised (29). This article presents the concept of new resistance-related parameters that differentiate mutants selected *in vitro* into dominant mutants (relevant in the public health context) and inferior mutants (unlikely to appear *in vivo* due to impaired fitness). We suggest the *in vitro*-determined MSW-D (the concentration range where dominant mutants are selected, limited by the MPC-D) should be avoided when adjusting doses *in vivo*, rather than the original MSW, which refers to all mutants selected *in vitro*. Moreover, this article shows that MPC-D can be lower than MPC. Thus, it should be more clinically useful due to the lower risk of side effects.

In this article, the MICs, MPCs, and SIs (MPC/MIC ratios) of ciprofloxacin, linezolid, and novel benzosiloxaborole No37 were determined for S. aureus ATCC 29213 by the initially proposed agar method (9). Values obtained for the parameters mentioned above were 0.25 mg per L/>8 mg per L/>32 for ciprofloxacin, 2 mg per L/4 mg per L/2 for linezolid, and 3.12 mg per L/100 mg per L/32 for compound No37. In the case of ciprofloxacin, the SIs reported so far for methicillin-susceptible Staphylococcus aureus (MSSA) strains with similar MICs to the strain tested in our study (0.12 to 0.5 mg/L) ranged from 8 to 64 (9, 23, 24). Such significant variations in the SIs of ciprofloxacin (6 to 156) have also been observed among wild-type Escherichia coli strains with similar MICs (25). In contrast, the linezolid SIs reported so far for S. aureus strains were usually equal to 2 or 4 (26, 39, 40). Likewise, no significant variability in the SIs of vancomycin (32 to 64) and fosfomycin (64 to >256) was observed among methicillin-resistant Staphylococcus epidermidis (MRSE) strains (27).

The initially proposed agar-dilution method for the MPC determination was successfully used by many authors, though it implicates several challenges. One is the so-called “inoculum effect” (41, 42). It is known that during MPC determination by agar-dilution assays, “false mutants” (with wild-type MIC) may appear, probably because huge inocula protect them from the drug action. Thus, testing the ability of obtained colonies to regrow on the same drug concentration is necessary (41, 42). Another way to eliminate this effect is to apply fewer cells on a large number of plates (33). This approach is time- and substance-consuming. Thus, it is hard to implement in preclinical screening, where the availability of new substances is frequently limited. In our study, we were also working with novel benzoxaborole No37, available in small amounts, and we had to minimize the plates’ usage. That is why we decided to eliminate the inoculum effect by performing MRTests instead of increasing the number of plates.

Obtaining the high-density inoculum is another challenge. Currently, required inocula are prepared via a one-step concentration of the liquid culture. However, they rarely exceed a density of 10^11^ CFU/mL (9, 36, 43), usually reaching 10^10^ CFU/mL (29, 39, 44 to 48) or 10^9^ CFU/mL (49, 50). The lower the inoculum density, the more plates are needed. Our multi-step concentration of the liquid culture, with many centrifugation-resuspension cycles, increases the chance of an inoculum with a density exceeding 10^11^ CFU/mL. Thus, it gives an advantage in circumstances when a substance is limited.

Finally, it was recently reported that MPC values sometimes show poor experiment-to-experiment repeatability, even within one laboratory. When determining the MPCs of 5 antibiotics from different classes for S. epidermidis was repeated 20 times, the results varied significantly for all tested agents (29). This is in contrast with the generally repeatable MIC values. We assume it is connected with the frequency of mutant selection. Mutants selected with low frequency may not always be present in the starting inoculum, as emphasized by Firsov et al. (51). This may make the upper boundary of the MSW changeable. We suggest that the FSMS value should provide a threshold for the MPC. As at least 1 × 10^10^ CFU must be tested for the MPC determination, we propose an FSMS of <1 × 10^−10^ (indicating less than 1 mutant per 10^10^ cells) for that. We consider that mutants selected less often are not the dominant mutants due to their rarity. Consequently, we proposed the lowest concentration with an FSMS of <1 × 10^−10^ as the new MPC-D parameter, defined as the lowest concentration that blocks the selection of dominant mutants. In our study, the MPC-Ds_10_^10^ of linezolid and No37 were equal to their MPCs. In the case of ciprofloxacin, however, the MPC-D_10_^10^ was at least 4-fold lower than the MPC_10_^10^ (16×MIC *versus* >32×MIC, respectively). At the highest concentration tested, mutants were selected with a frequency of 4.5 × 10^−11^. Thus, ciprofloxacin MPC determined in our study will not be reproducible in studies where less than 2.2 × 10^10^ CFU will be examined.

Regardless of the implicated challenges, the existence of the mutant selection window has already been confirmed in *in vitro* dynamic models. These models aim to simulate dosing regimens and cultivate bacteria in circulating broth with the tested agent, whose concentration fluctuates within the defined ranges (44). So far, for many strain–drug combinations, resistant mutants usually emerged when the drug concentrations were kept within the MSW range (39, 44, 46, 52, 53). However, some discrepancies occurred. Allen et al. isolated resistant mutants of S. aureus strains from a ciprofloxacin dynamic model, even when fluoroquinolones concentration was 2-fold higher than the designated MPC (23). On the other hand, the heterogeneity of the MSW was reported for the S. aureus strain with a broad ciprofloxacin MSW (SI = 16) (44, 53, 54). Authors noticed that its resistant mutants were enriched later and to a lesser extent when the drug concentration was kept in the upper compartment of the window than when it was kept in the lower compartment. Simultaneously, such differences were not observed for a strain with a narrow window. We consider this may be explained by the fact that mutants selected in the upper compartment are not the dominant mutants; in our study, the tested strain had wide ciprofloxacin MSW_10_^10^ (SI > 32), and its MPC-D_10_^10^ was at least 4-fold lower than its MPC_10_^10^.

It is also apparent that for many drug-strain combinations, the MSWs observed *in vitro* exist *in vivo* as well. This has been proven in the levofloxacin treatment of rabbits infected with S. aureus (20), E. coli (55), and Streptococcus pneumoniae (56), as well as for moxifloxacin tested in a murine model of tuberculosis (57) and for the marbofloxacin treatment of mice infected with E. coli (45). In all cases, animals received ~10^10^ CFU in 1 mL inoculum, and the treatment began when bacterial counts reached 10^8^ CFU/mL. Resistant mutants were generally recovered, and their numbers were found to increase during the treatment when the drug concentration fluctuated within the MSW. However, while testing fosfomycin against E. coli, the MSW was found to exist only *in vitro* (no mutants were recovered in a rabbit tissue cage model) (30), though it was associated with a decreased growth rate of the obtained mutants compared to the paternal strain, which probably made them easy to eradicate by the host’s immune system. Simultaneously, the fosfomycin MSWs for Pseudomonas aeruginosa were the same *in vitro* and *in vivo*, with no biological fitness costs of resistance occurred (30). On the other hand, Mei et al. confirmed no *in vivo* selection of the fosfomycin-resistant mutants of S. aureus ATCC 29213 within the *in vitro*-determined MSW boundaries, even though there was no decrease in growth rate for the mutants obtained *in vitro* (31). Such a result, however, may be associated with the immune system response and the pleiotropic fosfomycin action *in vivo*, which could not be predicted *in vitro*. Nevertheless, in some cases, *in vitro*-determined MSWs are broader than those observed later *in vivo*. We propose the broth-dilution method for the *in vitro* MPC determination to eliminate these discrepancies. In this method, the 24-h incubation of ~10^10^ CFU in a drug-containing broth allows the differentiation of selected mutants into dominant mutants (able to proliferate and establish a resistant population of at least 10 CFU/mL, i.e., able to dominate the population) and inferior mutants (unable to establish a resistant subpopulation due to impaired fitness). This is in contrast to the agar method, where mutants’ fitness does not influence the result. It was proven that the host’s immune system might successfully overcome mutants with impaired fitness and that they can be easily outcompeted by sensitive bacteria in a non-antibiotic environment (14, 15). In our study, the ciprofloxacin concentration that blocked the selection of dominant mutants was 4-fold lower than the concentration that blocked the selection of inferior mutants (MPC-D = 8×MIC *versus* MPC-F = 32×MIC). We consider inferior mutants unlikely to appear in *in vivo* studies. Thus, utilizing a dominant mutant selection window may increase compatibility with *in vivo* studies.

So far, few attempts have been made to determine the MPC value in a broth medium (42, 58 to 60). Quinn et al. tried to diminish the inoculum effect by increasing the volume of the liquid culture (e.g., introducing 10^10^ CFU into up to 1L of the drug-supplemented broth). However, it turned out that the inoculum effect affected that method too, though to a lesser degree (42). Other authors tested the modified microbroth-dilution method (microtiter assay) to facilitate the introduction of MPC testing into clinical laboratories, exposing up to 10^9^ CFU to tested drug concentrations. Obtained results were reproducible and comparable with the agar-dilution method (59, 60). In our study, however, we decided to expose 10^10^ CFU to each drug concentration (exactly as in the agar method), which corresponded to 100 μL of our inoculum. Thus, a microdilution assay was not possible to implement in this study. On the other hand, we also investigated a new compound with limited availability, so we had to minimize its usage. Finally, we decided to conduct our research by inoculating 5 mL of the drug-supplemented broth with 10^10^ CFU, whereas the inoculum effect was eliminated by testing the ability of obtained mutants to regrow on the same drug concentration (bMRTests). In our study, the MSW_10_^10^ ranges of linezolid and novel benzosiloxaborole No37 determined using the broth-dilution method were the same as those determined in the agar-dilution method. In contrast, the MSWs_10_^10^ of ciprofloxacin determined by the broth-dilution method were narrower (SI-D = 8, SI-F = 32) than the MSWs_10_^10^ determined in the agar-dilution method (SI-D = 16, SI > 32). However, the ciprofloxacin MPC-D_10_^10^ values determined in both methods were comparable (16×MIC in the agar-dilution method *versus* 8×MIC in the broth-dilution method). Thus, our results, like previous reports, indicate that the broth-dilution method may be utilized for MPC determination. It gives results consistent with the established agar-dilution method, and the compatibility is better for the MPC-D determination.

Finally, for the therapeutic application of the MPC values, the MPC-based pharmacodynamic parameter predicting the resistance development must be established (e.g., C_max_/MPC, AUC/MPC, or %T_MSW_). There is evidence supporting the hypothesis that MPC-based PK/PD indices may better predict resistance development than the corresponding MIC-based indices (61, 62). It was suggested that the mutants’ enrichment of the bacterial cell population depends not only on the total time the drug concentration is in the MSW range but also on its position within the MSW (52, 53). In other words, the time needed inside the MSW range for the mutants’ emergence and proliferation is shorter when the drug concentrations fluctuate within the lower compartment of the window (>30% when C_max_ < MPC) and significantly longer when they fluctuate in the upper compartment of the window (>80% when C_max_ > MPC) (20, 45). For other drug-strain combinations (e.g., marbofloxacin–K. pneumoniae), it is the ratio of the time (T) above the MPC value over the time inside the MSW range (T > MPC/T_MSW_) that seems to best predict resistance development (63). As no consensus has been reached, the MPC values are still not used in clinics. Consequently, improvement and standardization of the *in vitro* methods to determine the MPC value is crucial for further research, as it may facilitate establishing novel, MPC-D-related PK/PD indices as a basis for an “anti-mutant” dosing strategy. This, in turn, may prolong the life span of old and new antimicrobial agents.

### Conclusions.

The MPC is a parameter that was hypothesized to supplement the MIC value in the development of dosing regimens restricting the emergence of resistance. However, switching to high (MPC-based) doses, while those lower (MIC-based) are generally sufficient for a therapeutic success, is cumbersome. Moreover, MPC values show variability among strains with similar MICs, and concerns about their repeatability have been recently raised. Additionally, some discrepancies occurred between the MSW boundaries determined *in vitro* and those observed later *in vivo*. As a result, the MPC-based, “anti-mutant” dosing regimens are yet to be defined. This study proposes a new approach to the *in vitro* determination of MPC values and MSW ranges. It shows that these values may be lower than initially proposed when only related to dominant mutants (MPC-D and MSW-D). In the agar-dilution methodology, we propose determining the upper boundary of the MSW range based on the FSMS value (MPC-D instead of MPC) to increase the repeatability and reproducibility of obtained values. Second, we propose a novel broth-dilution method for determining the MSW range (marked MSW-D), which may eliminate the discrepancies between *in vitro* and *in vivo* values. Additionally, we propose a new method to achieve an inoculum that may exceed 10^11^ CFU/mL for a broader range of strains. Altogether, the proposed new approaches aim to facilitate establishing MPC-D-based, resistance-restricting dosing regimens and their clinical application.

## MATERIALS AND METHODS

### Antimicrobial agents, bacterial strain, and susceptibility testing.

S. aureus ATCC 29213 was used in this study. This strain is recommended by EUCAST and CLSI for the routine quality control of methods to determine the activity of antibiotics and chemotherapeutic agents against bacteria (21, 22). Before all experiments, the strain was stored at −80°C and subcultured twice on nutrition agar medium (BioMaxima SA, Lublin, Poland) at 35°C for 24 h to ensure viability. The following antimicrobials were tested: linezolid (Pol-Aura, Warsaw, Poland), ciprofloxacin (Pol-Aura, Warsaw, Poland), and novel benzosiloxaborole compound No37 (synthesized at Warsaw University of Technology, Warsaw, Poland) (64). Powdered forms of linezolid and ciprofloxacin were dissolved according to manufacturers’ instructions, while a stock solution of compound No37 was prepared in DMSO (Sigma, St. Louis, MO, USA). All stock solutions were used as fresh preparations. The susceptibility of S. aureus ATCC 29213 to the three tested compounds was examined by determining the MIC values according to the CLSI guidance, using the 2-fold broth microdilution method in Mueller-Hinton II broth medium (Becton, Dickinson and Company, Franklin Lakes, NJ, USA) (6). Strain susceptibility results were evaluated after incubation at 35°C for 18 h.

### Preparation of the high-density inoculum.

**FIG 2:**
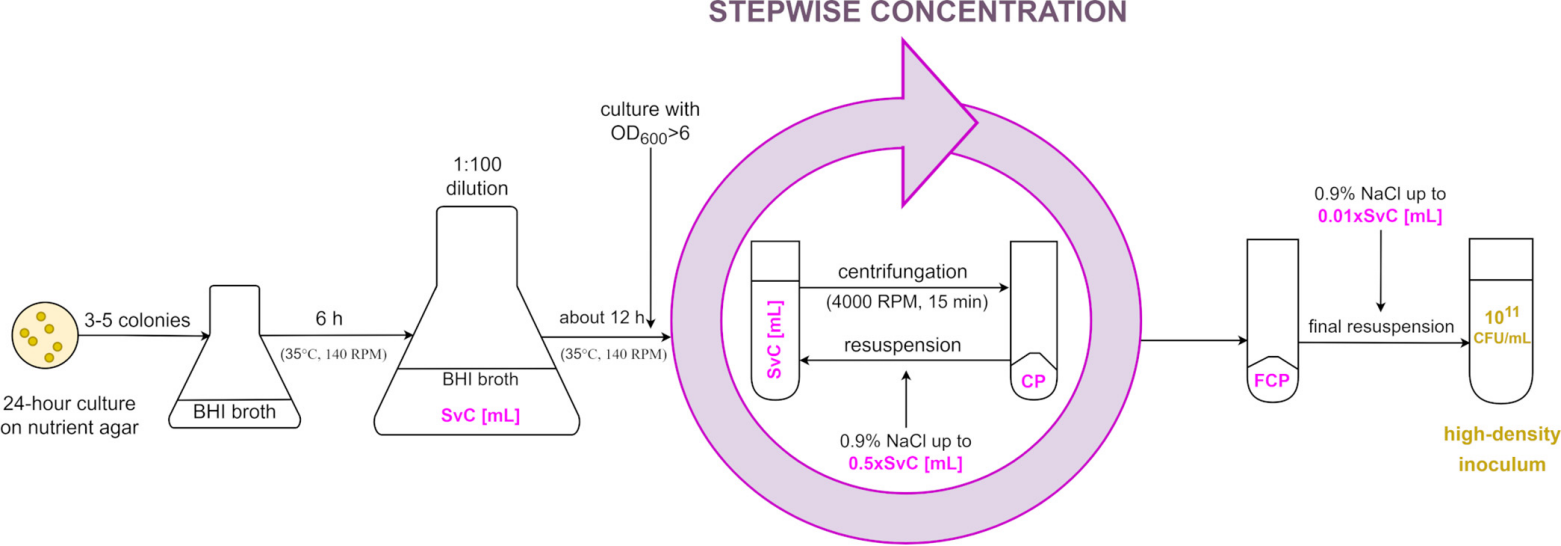
Preparation of the high-density inoculum. SvC, starting volume of bacterial culture; CP, cell pellet; FCP, final cell pellet; BHI, Brain Heart Infusion; 4,000 RPM = 2,061 × *g*.

The high-density inoculum (above 10^11^ CFU/mL) was obtained by multi-stage, progressive culture concentration by the centrifugation method (Fig. 2). First, three to five colonies of the tested strain from the 24-h culture on nutrition agar medium (BioMaxima SA, Lublin, Poland) were transferred to 15 mL brain heart infusion (BHI) broth (BioMaxima SA, Lublin, Poland) in a glass flask and incubated at 35°C for 6 h with shaking at 140 RPM. The obtained culture was then diluted 1:100 in fresh BHI broth in a glass flask and further incubated at 35°C for 12 h with shaking at 140 RPM. To ensure proper aeration, flasks were filled to a maximum of 50% of the flask volume. Cultures with OD_600_ of >6 were estimated to have a density above 10^9^ CFU/mL. If the OD_600_ values were below 6, the next measurement was performed after an additional hour of incubation. The culture with the required OD was centrifuged (2,061 × *g* = 4,000 RPM, 22°C, 15 min), and the obtained bacterial cell pellet was resuspended in sterile 0.9% NaCl to reach half the volume of the initial culture and recentrifuged (2,061 × *g* = 4,000 RPM, 22°C, 15 min). The procedure was repeated until the volume of obtained suspension was equal to 1/100 of the initial volume of the culture. This multi-step approach, with many centrifugation-resuspension cycles, ensured a proper washout of the medium, dead cell elements, and metabolites that might affect the viability of the final inoculum. The final inoculum was estimated to be above 10^11^ CFU/mL. The concentration of the final inoculum was confirmed by serial, 10-fold dilutions in 0.9% NaCl and plating on compound-free agar. After incubation at 35°C for 24 h, the bacterial colonies were counted, and the inoculum concentration was calculated.

### FSMS, MPC, MPC-D, MSW, and MSW-D determination by the agar-dilution method (AM).

**FIG 3:**
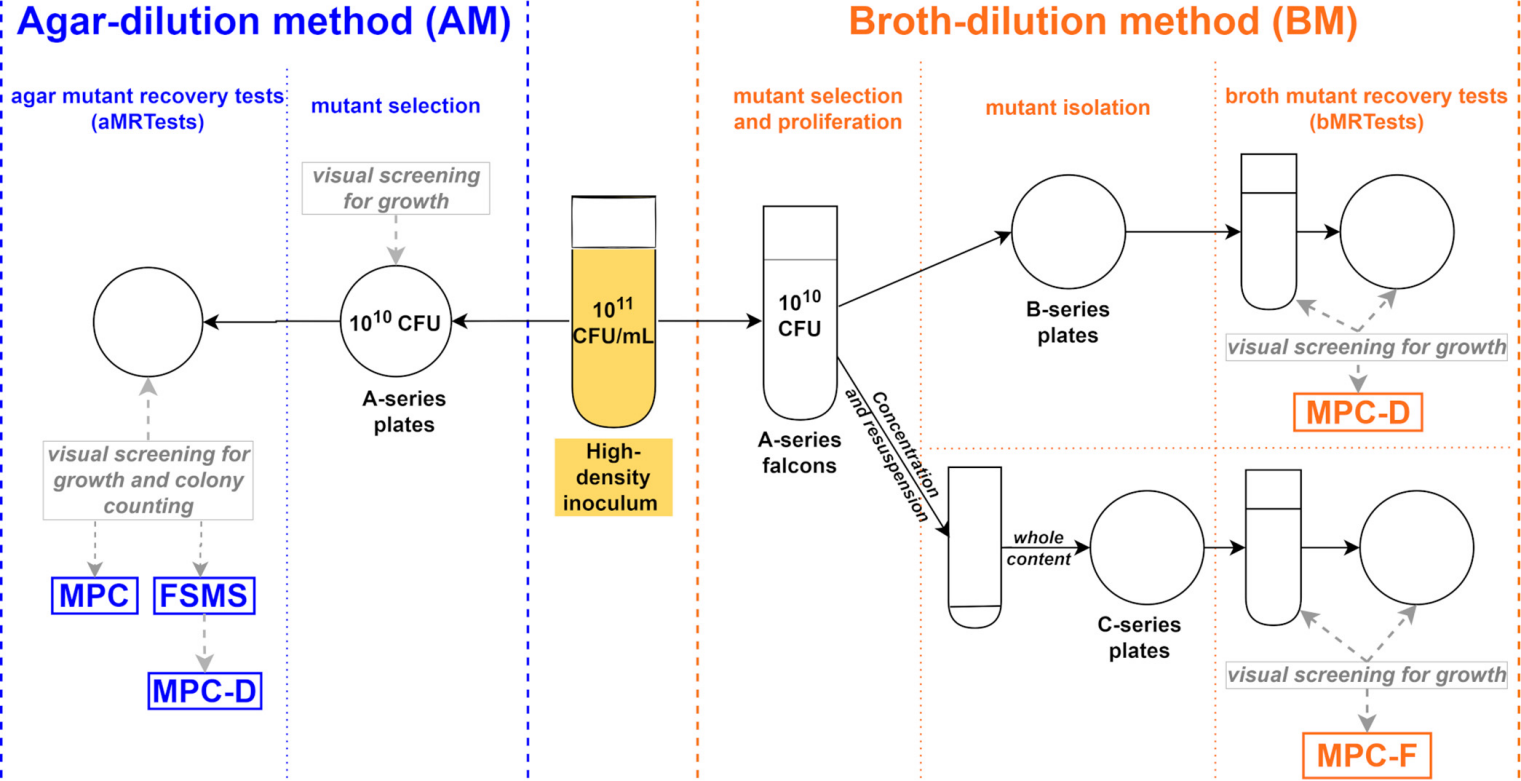
Comparison of the agar-dilution method and the broth-dilution method. MPC, mutant prevention concentration; FSMS, frequency of spontaneous mutant selection; MPC-D, dominant mutant prevention concentration; MPC-F, inferior mutant prevention concentration.

The FSMS, MPC, MPC-D, MSW, and MSW-D parameters were determined simultaneously by the AM (Fig. 3).

In the first stage of this study, a single-step resistant-mutant selection was performed. A series of MHII agar plates (Becton, Dickinson and Company, Franklin Lakes, NJ, USA) containing six 2-fold progressively increasing concentrations of the tested compounds (from 1×MIC to 32×MIC) were prepared. Four plates were prepared per concentration, and every plate was inoculated with 25 μL of the high-density inoculum (10^11^ CFU/mL) to ensure a total of 10^10^ CFU were exposed to each concentration. Aliquots were suspended in a 75 μL drop of 0.9% NaCl placed on the plate to facilitate the spreading of the high-density inoculum over the agar surface. All plates were incubated at 35°C for 24 h. They were designated A-series plates in which mutants were selected. After incubation, the plates were screened visually for growth, and colonies were counted. If no growth was visible, plates were reincubated for an additional 24 h under the same conditions. In the second stage of this study, the aMRTests were performed (9, 33). All obtained colonies, as well as the content of the visually clean plates and the content of plates with dense lawn, semi-dense lawn, and colony lawn were transferred to another agar plate with the same compound concentration. Plates were incubated at 35°C and screened for growth after 24 h and 48 h. No growth was confirmed after 72 h. Only colonies that regrew in the aMRTest were considered single-step resistant mutants while determining the FSMS, MPC, MPC-D, MSW, and MSW-D.

The MPC value was determined according to Dong et al. as the minimal concentration of the agent that causes no mutants to be obtained when a large number of cells (>10^10^ cells) are applied to agar plates containing the agent (9). The FSMS value for each concentration of tested agent was calculated as the ratio of the resistant CFU (mutant colonies grown on 32×MIC agar plates) to a total CFU in 1 mL of the initial inoculum. A frequency below 1 × 10^−8^ CFU/mL was considered the threshold for reduced mutant selection in the cell population (10, 13), whereas the lowest concentration of the agent with the FSMS value below 1 × 10^−10^ was taken as the MPC-D value. The lowest concentration that causes no mutant to be obtained on A-series plates was taken as the MPC value.

The MSW parameter was designated the interval of the agent concentration range from the MIC value up to the MPC value, whereas the MSW-D parameter was defined as the interval of the agent concentration range from the MIC value up to the MPC-D value.

Finally, the SI was calculated as the MPC/MIC ratio (10), while the SI-D was calculated as the MPC-D/MIC ratio.

### MPC-D, MPC-F, MSW-D, and MSW-F determination by the broth-dilution method (BM).

The MPC-D, MPC-F, MSW-D, and MSW-F parameters were determined simultaneously by the BM (Fig. 3).

A series of 2-fold progressively increasing concentrations of the tested compounds (from 1×MIC to 32×MIC) were prepared in MHII broth medium (Becton, Dickinson and Company, Franklin Lakes, NJ, USA). Each plastic laboratory falcon tube with 4.9 mL of MHII broth with the compound was inoculated with 100 μL of the high-density inoculum above 10^11^ CFU/mL. This provided an initial concentration of bacterial cells above 10^10^ CFU/mL in each falcon tube. Falcons were incubated at 35°C for 24 h with shaking at 140 RPM. They were designated A-series falcons, in which mutants were selected and proliferated. The mutants were then isolated by spreading two aliquots of 100 μL of the obtained culture over two MHII agar plates containing the same compound concentration as in the broth culture. These were designated B-series plates and incubated at 35°C for 48 h, then screened visually for growth, and the colonies were counted. To eliminate the inoculum effect, bMRTests were performed, during which the regrowth ability of the obtained resistant mutant colonies in the presence of the same concentration was checked, and the lack of growth on the agar mutant isolation plates was confirmed. Only colonies able to regrow in the bMRTest were taken into account. MPC-D was defined as the lowest drug concentration that, after the 24 h of incubation, prevents mutants selected among 10^10^ CFU from establishing a resistant population of at least 10 CFU/mL in the drug-supplemented broth culture (i.e., mutants are not able to dominate the population). This corresponded to at least 1 colony on each B-series plate. Mutants that established a population of at least 10 CFU/mL were named the dominant mutants.

To establish the MPC-F, the remaining content of the A-series falcons was centrifuged (2,061 × *g* = 4,000 RPM, 22°C, 15 min) and concentrated to the final culture volume of 100 μL. The entire content of each concentrated culture in the A-series falcons was transferred to MHII agar plates containing the same compound concentration as in the broth cultures. These were designated C-series plates and incubated at 35°C for 48 h, then screened visually for growth, and the colonies were counted. The obtained mutant colonies’ ability to regrow at the same concentration in broth, as well as the lack of growth on the agar mutant isolation plates, was examined by the bMRTest. Only colonies able to regrow in the bMRTest were taken into account for the determination of the MPC-F. The MPC-F was the minimal concentration of the agent that allowed no mutant to be obtained on the C-series plates. Mutants isolated on C-series plates that were not able to dominate the population were named the inferior mutants. The detection threshold for the MPC-F parameter determined by the BM is 1 CFU of mutant per 5 mL (the whole volume of the tested culture). This means that we can detect the presence of at least one mutant cell in 5 mL of the broth culture (which corresponds to 1 colony on the tested plates), where an initial concentration of bacterial cells was above 10^10^ CFU/mL in each falcon tube.

The MSW-D parameter was designated the interval of the agent concentration range from the MIC value up to the MPC-D value at which the agent inhibits the emergence of mutants capable of dominating the entire high-density population of the bacterial cells. The MSW-F parameter was defined as the interval of the agent concentration range from the MIC value up to the MPC-F value at which the agent inhibits selection, in a fluid environment, of resistant mutants appearing in the entire population of high-density bacterial cells.

Finally, the SI-D was calculated as the MPC-D/MIC ratio, while the SI-F was calculated as the MPC-F/MIC ratio.

### Broth mutant recovery test (bMRTest).

The in-broth recovery test was performed for resistant mutants obtained on the B- and C-series plates. The obtained mutant colonies on the plates with compound, the content of the plates on which a dense lawn, a semi-dense lawn, or a colony lawn appeared, and the content of the visually clean plates were transferred to MHII broth with the same compound concentration. falcons were incubated at 35°C with shaking (140 RPM) and were visually screened for growth after 24 h, 48 h, and (if no growth was visible) after 72 h. The observed turbidity of the broth medium indicated the growth of bacteria. To confirm the regrowth ability of the obtained resistant mutants in the presence of the antibacterial compound, a sample of this turbid culture from the falcon tube was plated on agar supplemented with the same compound concentration and incubated at 35°C for up to 72 h. A sample of the culture from the falcon tube that was visually clear after 72 h of incubation was transferred to the MHII agar plate with the same compound concentration and incubated at 35°C for up to 72 h to confirm the lack of growth.
